# CT Image Sequence Restoration Based on Sparse and Low-Rank Decomposition

**DOI:** 10.1371/journal.pone.0072696

**Published:** 2013-09-04

**Authors:** Shuiping Gou, Yueyue Wang, Zhilong Wang, Yong Peng, Xiaopeng Zhang, Licheng Jiao, Jianshe Wu

**Affiliations:** 1 Key Lab of Intelligent Perception and Image Understanding of Ministry of Education, Xidian University, Xi’an, Shaanxi,China; 2 Radiology Department of Xijing Hospital, The Fourth Military Medical University, Xi'an, Shaanxi, China; 3 Beijing Cancer Hospital, Peking University School of Oncology, Beijing, China; Virginia Tech, United States of America

## Abstract

Blurry organ boundaries and soft tissue structures present a major challenge in biomedical image restoration. In this paper, we propose a low-rank decomposition-based method for computed tomography (CT) image sequence restoration, where the CT image sequence is decomposed into a sparse component and a low-rank component. A new point spread function of Weiner filter is employed to efficiently remove blur in the sparse component; a wiener filtering with the Gaussian PSF is used to recover the average image of the low-rank component. And then we get the recovered CT image sequence by combining the recovery low-rank image with all recovery sparse image sequence. Our method achieves restoration results with higher contrast, sharper organ boundaries and richer soft tissue structure information, compared with existing CT image restoration methods. The robustness of our method was assessed with numerical experiments using three different low-rank models: Robust Principle Component Analysis (RPCA), Linearized Alternating Direction Method with Adaptive Penalty (LADMAP) and Go Decomposition (GoDec). Experimental results demonstrated that the RPCA model was the most suitable for the small noise CT images whereas the GoDec model was the best for the large noisy CT images.

## Introduction

Accurate display of anatomical detail, in the form of small structures, features and objects, is a key requirement of computed tomography (CT). These details carry important information that may be associated with distinction between normal and pathological diagnosis. However, blurring of the CT images often compromises the visibility of such fine details.

Generally, CT modality is an economical way to generate high-resolution images of human anatomy. CT images show more complex details of bones structures, compared to magnetic resonance imaging (MRI) images. However, one side CT images are degraded by blurring due to many reasons, such as the imperfect resolution of the imaging system, data loss in acquisition, and acquisition noise, to name a few. These artifacts and data loss result in weak contrast and blurry organ boundaries. Furthermore, streak artifact around thick cortical bone results in degraded visibility of some soft tissue tumors present in low contrast regions. Therefore, contrast enhancement for CT images is critical to facilitate evaluating the extent of disease in soft tissue area [1]. Another, source of degraded CT image is the presence of noise, which adds further blur to organ boundaries.

Recently, many CT image deblurring methods have been studied to visualize miniature-sized features. Jiang et al proposed a deblurring method for spiral CT image based on edge signal-to-noise ratio in 2003 [2]. Their method obtained the standard deviation
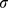
of two-dimension Gaussian blur kernel by calculating the minimum value of the edge signal-to-noise ratio of the blurred CT image, and then deblurred the CT image using the EM algorithm. This deblurring method significantly improved the identification of cochlear CT details. In 2005, Wang made an improvement to Jiang's method using the Wiener filter in place of the EM algorithm [3], and accelerated the speed of deblurring algorithm without sacrificing the deblurring effect. Later, based on edge-to-noise ratio and constrained least squares, Zhou proposed a filter blind deblurring algorithm and an iterative blind deblurring algorithm respectively [4]. Both of them significantly improved the image quality, compared with Jiang's and Wang's methods. In 2010, Hussien also used the Wiener filter to restore CT soft tissue [1]. In 2012, Zohair presented a fast deblurring method for CT medical images using a novel kernel set [5].

All the deblurred methods mentioned above only deal with a single CT image without considering the context of image sequence. these methods can also deal with CT image sequences via one by one, which just likes to deblur repeat single image several times with the same operations and the information between adjacent frames are not considered. There may be time-consuming. In this study, we will explore a new image sequence deblurred algorithm by using sparse representation and low-rank decompose model. For the reason that we always obtained CT image sequences from CT medical imaging system and the doctors judge the state of illness according to the changes of the CT image sequence, so we think that dealing with CT image sequence is more significant than dealing with a single CT image. Our method is also suitable for a single CT image, the difference is that we deblur the low-rank component working out the average low-rank image for sequence image but on own rank of a slice CT for single image. The result may slightly worse than using the average rank of image sequence if sequence image have big difference from slices. It is the fact that a single image can use information between adjacent slices. Recently, low-rank technology is used to decompose image sequence into a low-rank component and a sparse component. In low-rank model, noise and blur information is considered to exist in the sparse component of decomposition image, whereas original image information exists in the low-rank component [6]. Following a similar philosophy, CT sequence images can also be decomposed into the sparse and low-rank component, and restoration can be applied to the sparse and low-rank components, respectively. In this paper, a CT sequence image restoration algorithm is proposed based on low-rank decomposition. Three classic low-rank models have been assessed in the experiments; and comparisons were performed on different medical images.

## Review

A CT medical imaging system can be modeled as a linear transformation of an incoming analog signal, which is corrupted by intrinsic measurement fluctuations or electronic noise. A blurred image 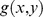
is assumed to be generated by convolving the latent image 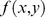 with a blur kernel 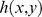
with a latent image, with additive noise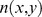:



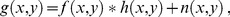
(1)where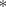 denotes convolution operator [7]–[10].

The main cause of degradation in medical CT images is the imperfect resolution of the imaging system, and the degradation function of a two-dimensional medical CT image is generally approximated as an isotropic two-dimensional Gaussian Point Spread Function (PSF) [11]:



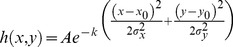
(2)where
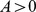
is a constant, 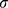
is the variance.

Image restoration technique aims to recover an image from its observation, degraded with blur and noise. In order to restore the image, prior knowledge of the degradation and inverse filtering must be obtained. Direct inverse filtering is usually used. Unfortunately,it is invalid when noise is taken into consideration. Another, degradation process typically happens in CT medical imaging, which includes blurring caused by imperfect resolution of the imaging system and noise by random fluctuations in signal intensity. Therefore, Wiener filtering [12]–[14] is a better choice than inverse filtering since it takes noise into consideration for inverse filtering.

We can obtain the expression of the formula
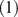
in the frequency domain through the discrete Fourier transform:



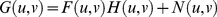
(3)where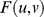,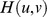,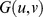 and 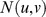 are the discrete Fourier transform of the latent image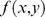, blur kernel 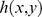, blurred input image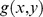 and noise 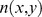 respectively. Wiener filtering restoration is a method that regards 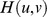 as its transmission function, and generates the recovery image 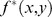 by minimizing the mean square error between the recovery image 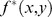
and the latent image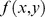, namely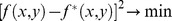. The expression of the recovery image by wiener filtering is



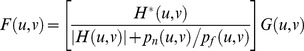
(4)where
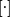
denotes wiener filtering, 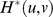 is the conjugate matrix of 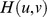,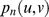 and 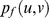 are the power spectrums of noise and latent image respectively. It is often hard to estimate the values of 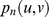
and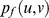, and we use the following expression to approximate the wiener filtering restoration.



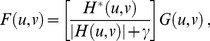
(5)where
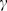
is a constant, which is numerically takes the reciprocal value of the signal-to-noise ratio of the blur image. The recovery image can be further calculated according to equation 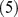
by the Fourier inverse transform: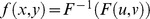.

## Our Method

### 1. Ethics Statement

This study was approved by Key Lab of Intelligent Perception and Image Understanding of Ministry of Education and by the institutional review board (IRB) of The School of Oncology, Peking University (Beijing Cancer Hospital). The doctors obtained signed informed consent forms from all selected patients prior to the routine clinical course of CT examinations.

### 2. Motivation

**Figure 1:**
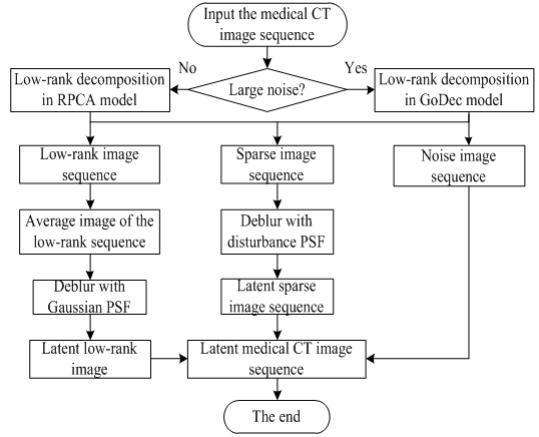
Flow chart of our algorithm.

Generally, we obtained CT image sequences from CT medical imaging system, but the existing deblurred methods always focus on the single image. In fact, each CT image in the same sequence is similar with its adjacent frames or maybe with a little change. Another, it was shown that a low-rank matrix could be recovered from incomplete sampling of its entries by computing the matrix of minimum nuclear norm the data [15]. Inspired by the image decomposition in Ref. [6] and the video processing, CT image sequences are decomposed by sparse and low-rank here. We can find noise and blur information exist in the sparse component of decomposition image, whereas most of the original image information exists in the low-rank component. Thus, CT sequence images restoration can be operated both in sparse and low-rank components, respectively. Now, sparse and low-rank technology mainly includes three kinds of low-rank decomposition models, and each low-rank model has its emphasis. In this study, both RPCA and GoDec models will be used to deblur CT image sequences. More details can be seen from the following subsections. The frame of our work is shown in Figure 1.

### 3. Framework of sparse and low-rank matrix decomposition

Sparse and low-rank decomposition is widely used in background modeling and shadow removal; it usually focuses on image sequences due to their intrinsic sparse characteristics. Medical CT image sequence has strong correlation between adjacent frames, and it is pointed out that medical CT images are sparse [16], Inspired by the image decomposition in natural image deblurring, we applied the sparse and low-rank decomposition for CT image deblurring. In this section, we will briefly introduce the image decomposition with three low-rank models.

#### Model 1


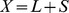
 **,** where
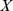
is the data matrix of the original image,
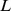
is a low-rank matrix, 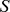
is a sparse matrix.

By using the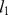-norm as the proxy of sparsity and the nuclear norm as the surrogate for low-rank, as in statistics and image processing [15]-[18], the sparse and low-rank recovery can be accomplished by solving the following RPCA problem:



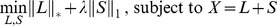
(6)where
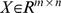
is the data matrix of the original image,
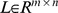
is the low-rank component of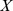,
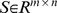
is the sparse component of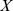, 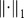
is the 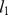-norm defined by the component-wise sum of the absolute values of all entries,
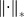
is the nuclear norm defined by the sum of singular values, 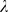
is a positive weighting parameter. This problem arises in many applications, such as image processing, web data ranking, and bioinformatic data analysis. Under surprisingly broad conditions, the RPCA problem can be exactly solved via convex optimization that minimizes a combination of the nuclear norm and the 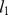-norm. It has the advantages of high speed and precision. For the RPCA problem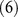, we may apply the augmented Lagrange multiplier method by identifying:



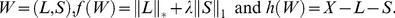
(7)


Then its Lagrangian function [19]–[22] is:



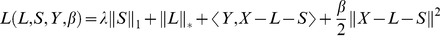
(8)where
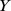
is the Lagrange multiplier, 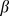
is a positive scalar, 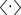
denotes the standard trace inner product and 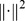
is the Frobenius norm. Its iterative scheme is:



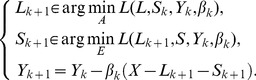
(9)


Formula 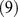
can be solved by the following:



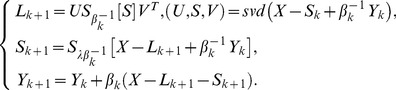
(10)


This algorithm solves the RPCA problem through the Lagrange multiplier method, which recovers a low-rank matrix with an unknown fraction of its entries arbitrarily corrupted.

#### Model 2


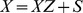
, where
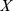
is the data matrix of the original image, 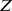
is the skinny SVD, 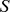
is a sparse matrix.

The decomposition problem based on model 2 can be converted into the following optimization problem:



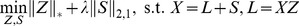
(11)


Its augmented Lagrangian function [23]–[28] can be written as the following equation:



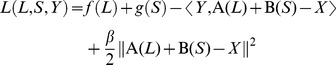
(12)where 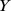 is the Lagrange multiplier, 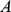
and 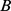 are linear mappings, 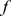
and 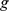
are convex functions,
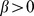
is the penalty parameter, 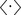 denotes the inner product, 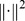 is the Frobenius norm. Its iterative scheme is:



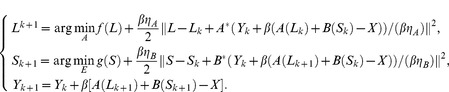
(13)where
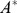
is the adjoint of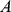,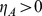 and 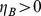
are parameters. Penalty parameter
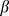
is updated according to the formula: 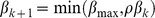, where
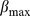
is an upper bound of 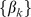. The value of
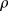
is defined as



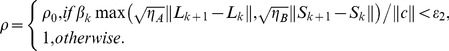
(14)where
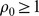
is a constant.

This is the LADMAP algorithm. It solves the low-rank representation problem through the alternating direction method, and linearizes the quadratic penalty term adaptively. Compared with the traditional alternating direction method, this method has a novel rule to update the penalty so that it converges fast, without the need to introduce auxiliary variables or to invert matrices during the operation process. It has found wide applications in computer vision and machine learning.

#### Model 3


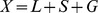
,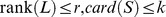, where
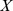
is the data matrix of the original image,
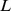
is a low-rank matrix, 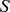
is a sparse matrix, 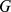
is a noise matrix, 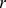
is the rank of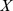, 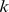
is the candinality. During the decomposition process, the algorithm alternatingly assigns the 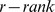
approximation of
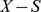
to
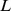
and assigns the sparse approximation with cardinality
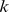
of
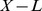
to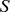.

The decomposition problem based on model 3 can be converted into the following optimization problem [29]–[34]:



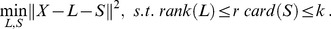
(15)


Formula 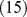 can be converted into the following two sub-problems:



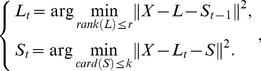
(16)


which is equivalent to



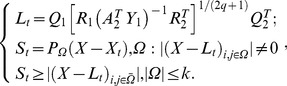
(17)where 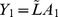,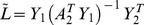,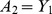;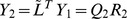, 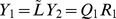,
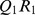
and 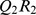
are the QR-decomposition of 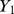
and 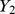
respectively.

This is the GoDec algorithm, which is a low-rank approximation method based on bilateral random projections. The algorithm can approximate the low-rank component, the sparse component and the noise component of the input data matrix. It has the advantage of small relative error and high decomposition speed.

To sum up, RPCA and GoDec share similar motivations in that they both explore the low-rank and sparse structures in the given data matrix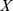. They differ, however, by their distinctive ways of handling the specific decomposition but they are intrinsically different. RPCA assumes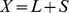 (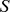
is sparse noise) and exactly decomposes
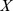
into
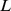
and
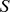
without pre-defined rank(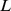) and card(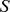), that is, RPCA offers a blind separation of low-rank data and sparse noise, however, GoDec produces approximated decomposition of a general matrix 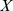
whose exact RPCA decomposition does not exist due to the additive noise 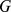
and pre-defined rank(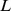) and card(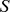); GoDec directly constrains the rank range of 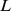
and the cardinality range of 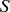, while RPCA minimizes their corresponding convex polytopes (i.e., the nuclear norm of 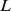
and 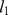-norm of 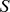); GoDec usually produces less relative error with much less CPU seconds than RPCA. And the improvement of accuracy for the model of GoDec is due to more general than that of RPCA by considering the noise component. LADMAP and RPCA are same intrinsically, but LADMAP reduces the auxiliary variables and constraints, further accelerates the convergence speed and improves the accuracy of decomposition.

### 4. Restoration using low-rank matrix decomposition on CT image sequence

In this paper, sparse and low-rank matrix decomposition was applied to the problem of medical CT image sequence restoration. For the blurred CT image, it is necessary to recover its low-rank component, because it conveys major information on the latent image. The sparse component, on the other hand, normally reflects the majority of blurring. In practice, CT images differ in their characteristics: some contain high level of noise that seriously degrades image quality and affects visualization; in others, the noise level is relatively low and has little impact on the visual quality of the image or diagnosis of the lesions. Therefore, it is necessary for us to select the appropriate low-rank model based on the image characteristics, and to perform low-rank decomposition and restoration in order to obtain the desirable recovery results.

We propose a new CT image sequence restoration model based on low-rank decomposition as follows:



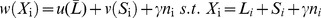
(18)where
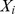
is the 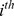 slice of the original CT sequence, 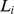
is a low-rank matrix, 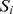
is a sparse matrix, 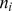 is a noise matrix of 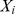, 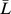
is the average of all the low-rank matrix 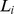,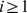, 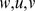 are recovery functions, 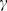
is 0 or 1 according to the low-rank model.

Also, there exist several methods using the information of the adjacent frames, such as the methods introduced in ref. [35], [36], but they use the idea that the corrupted portions of the projection data can be substituted with the corresponding portions from an unaffected adjacent slice, and restore the image one by one. Our method can restore the image sequence by one-time processing, which can not only consider the common characters of the image sequence but also take the different features of each image into account.

In natural imaging, the image blur can be regarded as atmospheric turbulence affecting the observed scene captured by the camera due to fluctuations of the refraction index of the medium [37]. This phenomenon may also exist in the medical CT imaging process caused by the refraction of the human tissue when the X-ray go into the human body, so we propose another degradation function we call turbulence PSF in this paper, which is acted on the sparse component for wiener filtering, the expression is as follows [38]:



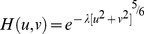
(19)where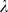 is a constant.

This degradation function we provided was inspired by the blurring natural image and then we think the turbulence also exist in CT imaging based on the reference [11]. Furthermore, after CT image sequences are decomposed into sparse and low-rank components, blur information has often concentrated on the sparse component of decomposition image and there is not much difference from low-rank components. Therefore, turbulence PSF is acted on the sparse component as well as Gaussian PSF is used on the average low-rank component. In order to demonstrate the performance of provided degradation function, we did some comparison experiments that we deblur a single CT image with only turbulence PSF, only Gaussian PSF and both the two PSFs, the result shows that use both the PSFs have more advantages.

The detailed steps of our algorithm are as follows:

**Algorithm:** CT image sequence restoration based on sparse and low-rank decomposition

**Input:** the blurred CT image sequence

**Output:** the latent CT image sequence

**Step 1:** Input the medical CT image sequence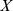. 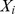
represents the
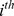
image in the sequence 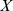,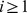. If
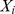
is acolor image, convert it to gray scale;

**Step 2:** Use all the CT images in the sequence
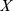
to synthesize a high dimensional matrix, each column of the high dimensional matrix represents a single CT image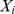;

**Step 3:** select a reasonable low-rank model to decompose the high dimensional matrix in step 2 into high dimensional sparse matrix, low-rank matrix and noise matrix according to the actual situation of the CT image sequence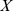;

**Step 4:** convert the high dimensional sparse matrix, low-rank matrix and noise matrix into sparse image sequence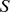, low-rank image sequence
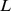
and noise image sequence 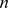, respectively;

**Step 5:** Using our restoration model 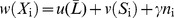
to restore the blurred CT image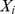, where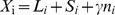. 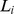,
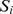
and 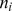
are the
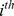
images of image sequence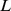,
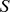
and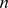,respectively. For each slice image, when Gaussian PSF acts on 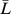, common knowledge of sequence images is used to restore a low-rank matrix of 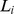 whereas sparse blurring component uses own 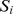 matrix to restore by turbulence PSF.

### 5. Evaluation of Recover

In order to objectively evaluate the performance of the image restoration algorithm, we use standard deviation, image information entropy and image quality measurement function value [39]–[41] as evaluation indices for comparison in this paper.

The formula of standard deviation is as follows



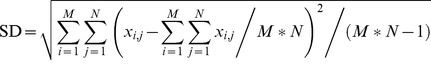
(20)where 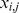
denotes the pixel value of location 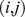
of the recovery image, and the size of the recovery image is 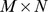.The greater the standard deviation value, the more dispersed the gray level distribution, the greater the image contrast, and the more detailed information.

Information entropy is one of the key indicators to measure how much image information is: the greater the value, the larger the amount of information contained in the image, its expression is



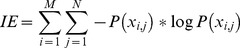
(21)where 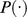
denotes the probability.

Image quality measurement function value is defined as



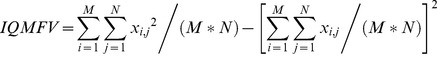
(22)


The greater the image quality measurement function value, the more uniform the gray level distribution, and the better the image quality.

## Experiments and Analysis

Based on the methods we described in Section 3, we investigate the most appropriate low-rank models for various types of CT images, differing in their noise levels and properties. Each image in our CT image sequences is 512 by 512 in size, with RGB color, obtained on a Discovery CT750 HD at the Beijing Cancer Hospital. We used 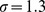 in the Gaussian PSF, and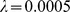 in the turbulence PSF for our experiments. All methods were implemented in Matlab7.10.0 (R2010a), running on desktop with an Intel Core i3 CPU at 2.20GHz and 2GB memory, employing a 32-bit windows 7 operating system.

### A. Comparison of different low-rank models

**Figure 2:**
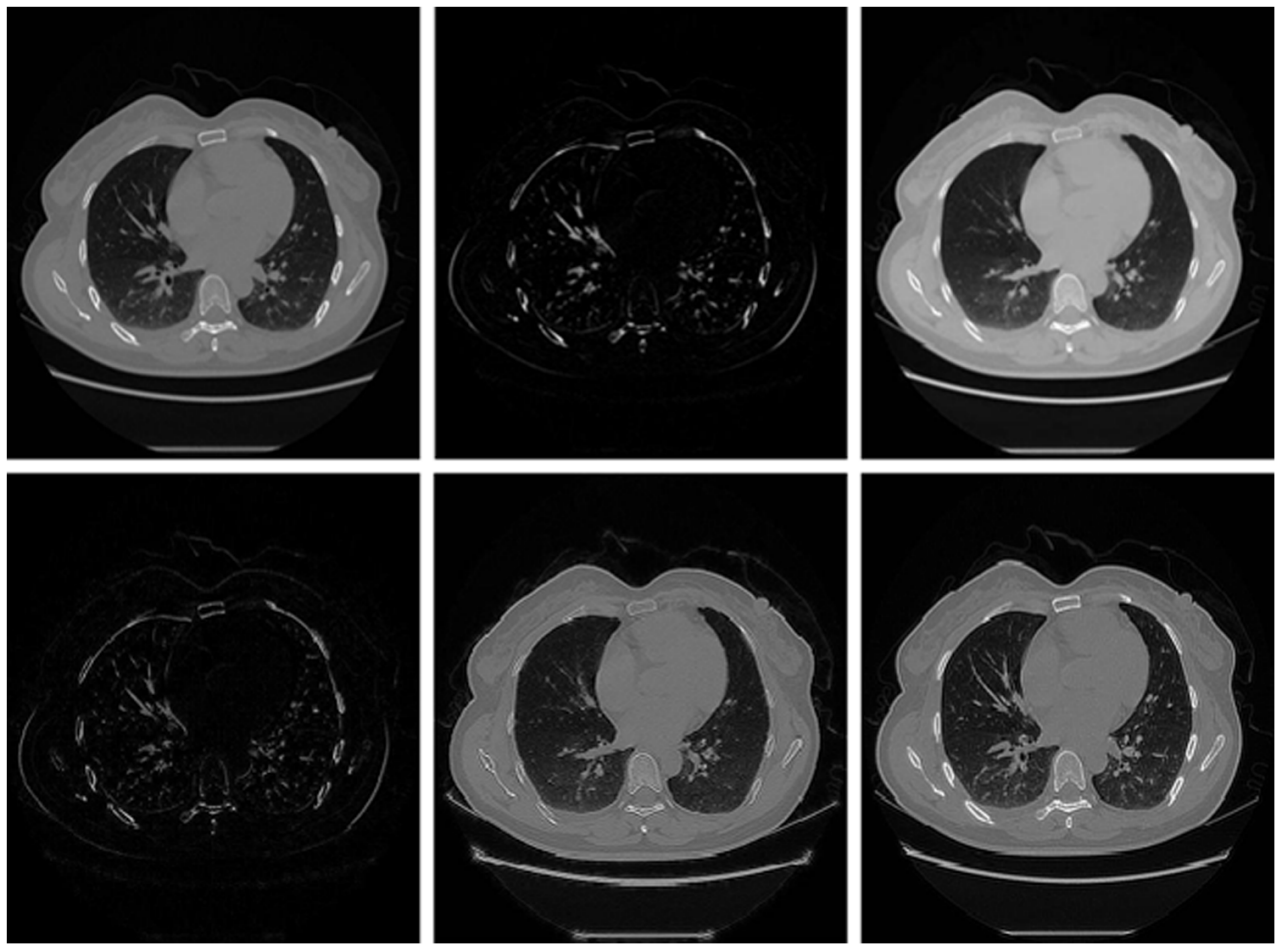
Shows restoration procedure of sparse and low-rank decomposition. From left to right, from top to bottom: the first image of the chest CT image sequence, the sparse component, the low-rank component, the recovery image of the sparse component, the recovery image of the low-rank component and the recovery CT image. We can see that the detailed information and the visual effect of the CT image after restoration are obviously improved.

We first use the chest CT image sequence to evaluate the contrast of different low-rank models. The noise is small on each image of the CT sequence and does not affect the basic visualization. We decompose the input chest CT image sequence into a sparse component and a low-rank component in the RPCA model, and then recover the two components using wiener filtering, we use
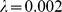
in this experiment, and the decomposition criterion is to keep all the low-rank component as much as possible, the procedure is shown in Figure 2.

**Figure 3:**
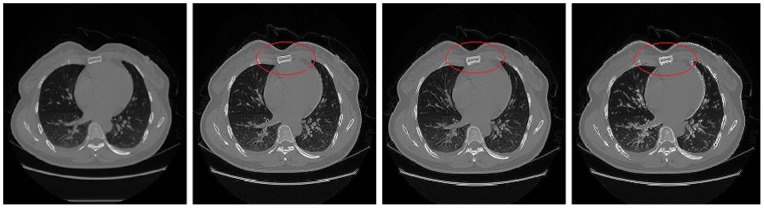
Shows comparison of restoration effect of three low-rank models in dealing with the noiseless CT images. From left to right: the 10^th^ original chest CT image, the result of RPCA model, the result of LADMAP model, the result of GoDec model.We can see that for noiseless CT image, our method can get satisfactory results, but ringing effect of the recovery image based on the RPCA model is much smaller than the other two models, its advantage in visual effect especially shown in the component which is circled with red ellipses in the images.

In order to analyze the scope of application of the three different low-rank models, we applied all decomposition models to the same chest CT image, and restored the chest CT image sequence. To illustrate the effectiveness of the proposed method in recovering the CT image sequence, the 10^th^ original image of the chest CT image sequence and its recovery images in different models are shown in Figure 3. For RPCA and LADMAP, we used
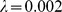
and
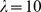
respectively, and for GoDec,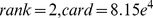.

To further compare the recovery effect of the three models, we list in Table 1 the image evaluation index values mentioned above for every image in Figure 3, as is shown.

**Table 1:** Comparison of evaluation index values of the recovery images of the three models.

	SD	IE	IQMFV
The 10^th^ CT image	49.2172	5.5966	2422.3
RPCA model	55.0057	**6.1122**	3025.6
LADMAP model	54.4365	6.0759	2963.3
GoDec model	**57.1869**	6.0368	**3270.3**

From Table 1, we can see that the recovery images using our method have great improvement in image standard deviation, information entropy, and quality measurement function value, compared with the original chest CT image. Although the image standard deviation and quality measurement function value of GoDec model is much greater, indicating sharper contrast, the recovery image has more serious ringing effect. Therefore, for the medical CT image sequence containing a low level of noise, the RPCA model is preferable to decompose the sequence, resulting in better recovery results.

**Figure 4:**
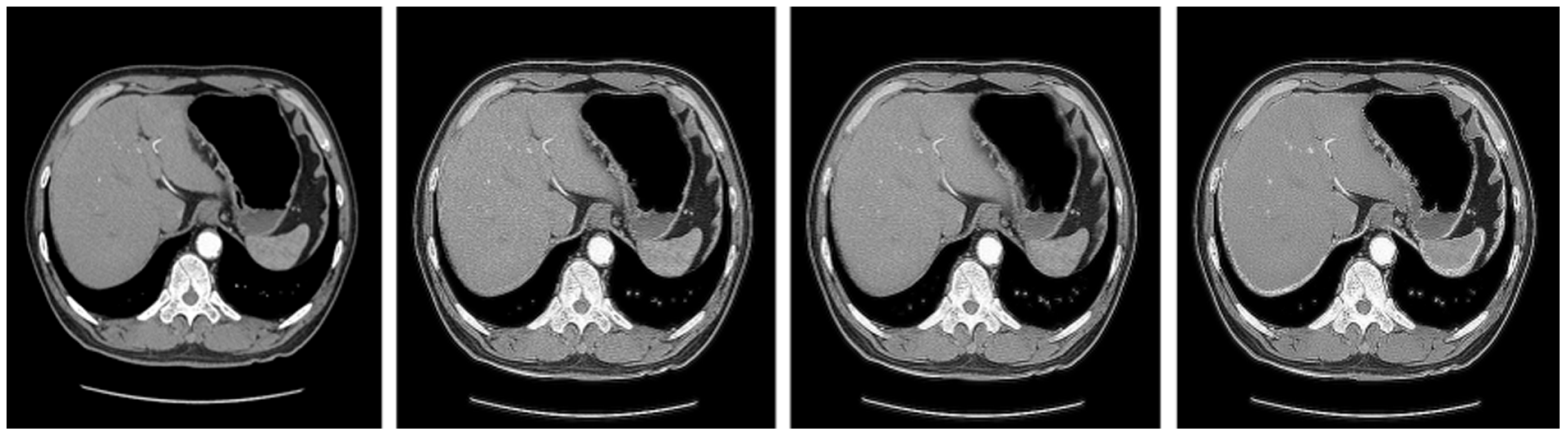
Shows comparison of restoration effect of three low-rank models in dealing with the CT images that contain large noise. From left to right: the 10^th^ original stomach CT image, the result of RPCA model, the result of LADMAP model, the result of GoDec model, we can see our method can do some good effect on the medical CT images which contain large noise, the contrast of the images after restoration enhance obviously and the human organ boundaries are clear, there is more detailed information shown out, but the recovery image based on GoDec model shows less noise than the other two images.

The same comparative experiments were also performed on a stomach CT image sequence, which has a much larger noise component. The results of comparison are shown in Figure 4 and the corresponding evaluation index values of every image are shown in Table 2. For RPCA and LADMAP, we used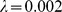 and 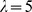
respectively, and for GoDec, 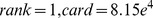.

**Table 2:** Comparison of evaluation index values of the recovery images of the three models.

	SD	IE	IQMFV
The 10^th^ CT image	68.0978	3.9737	4637.3
RPCA model	75.8182	4.7177	5748.4
LADMAP model	75.1148	**4.7347**	5642.2
GoDec model	**76.3726**	4.6915	**5832.7**

From Table 2, we can see the recovery images using our method have a great improvement in image standard deviation, information entropy and quality measurement function value compared with the original CT image. Although the difference among the evaluation index values of the three recovery images is small, the recovered image based on the GoDec model shows less noise than the results from the other two methods. Therefore, for the CT images with large noise, the GoDec model is more preferable to decompose the image sequence.

### B. Comparison of different recovery methods

**Figure 5:**
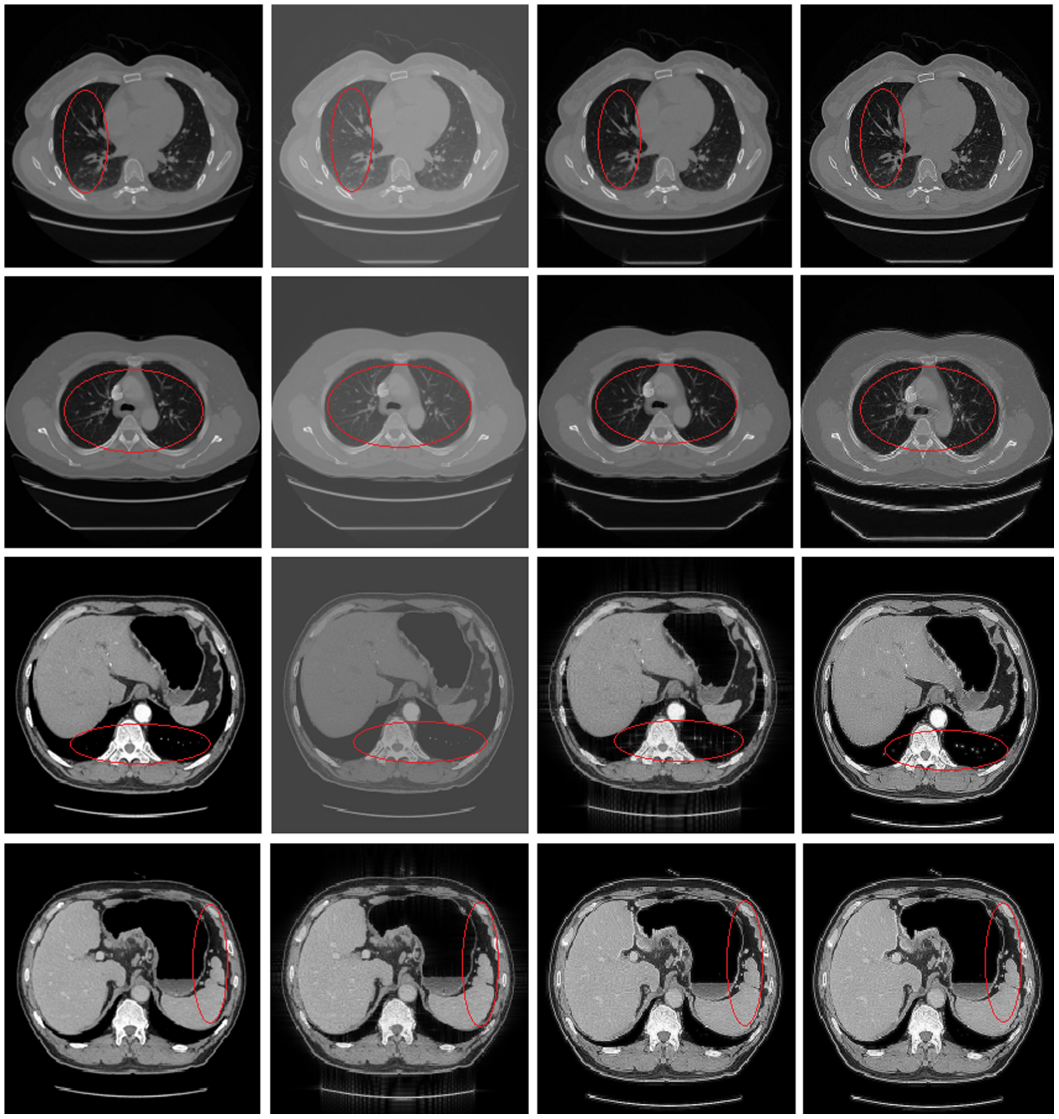
Shows comparisons of different methods. From top to bottom: chest CT experiment, lung CT experiment, stomach CT (1) experiment, stomach CT (2) experiment. From left to right in each row: the original image, the recovery image using Wang's method, the recovery image using Hussien's method, the recovery image using our method. It needs to say, we select the RPCA model in chest CT and lung CT, and select the GoDec model in stomach CT. We can see that using our method to restore the medical CT image sequence can obtain satisfactory results, the recovery images can show high contrast, clear detail information and clear organ boundaries which are especially shown in the components circled with red ellipses in the images.

To illustrate the effectiveness of the proposed method, we make several groups of contrast experiments between different methods which are shown in Figure 5. We list the image evaluation index values of every group above in Tables 3–6.

**Table 3:** Comparison of evaluation index values of the chest CT image.

	SD	IE	IQMFV
Original CT image	50.3358	5.5876	2533.7
Wang's [3]	50.2142	5.2891	2521.5
Hussien's [1]	50.4130	5.7766	2541.5
ours	**54.5948**	**6.0645**	**2980.6**

**Table 4:** Comparison of evaluation index values of the lung CT image.

	SD	IE	IQMFV
Original CT image	47.2274	5.5265	2230.4
Wang's [3]	46.7205	5.2103	2182.8
Hussien's [1]	48.6962	5.8733	2371.3
ours	**54.3555**	**6.0751**	**2954.5**

**Table 5:** Comparison of evaluation index values of the stomach CT image (1).

	SD	IE	IQMFV
Original CT image	71.1016	4.2681	5055.4
Wang's [3]	71.9176	4.9294	5172.1
Hussien's [1]	67.9674	**4.9868**	4619.5
ours	**74.0204**	4.6054	**5479.0**

**Table 6:** Comparison of evaluation index values of the stomach CT image (2).

	SD	IE	IQMFV
Original CT image	71.1016	4.2681	5055.4
Wang's [3]	71.9176	4.9294	5172.1
Hussien's [1]	71.3398	**5.1978**	5089.3
ours	**78.4211**	4.8226	**6149.9**

For the chest CT image comparative experiment, we used variance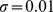, noise-to-signal 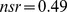
for Wang's method, and used
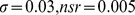
for Hussien's method. The decomposition parameter in our method were 0.002,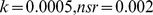. For the lung CT image comparative experiment, we used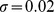, 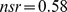
for Wang's method, and 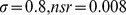 for Hussien's method. In our method, the decomposition parameter were 0.0025,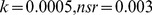. For the stomach CT image comparative experiment, we used 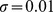, 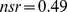 for Wang's method, and 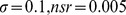 for Hussien's method. In our method, the decomposition parameters were 8.15e^4^,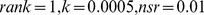.

The four groups of comparison of evaluation index values reveal that our method have advantages in all the evaluation index values when deal with the low level noise CT image. But the information entropy values of our method is lower than some of the others in case of high level noise CT images. What we want to clear is that there is no more other effective evaluation index to address this problem so far, So we used information entropy to try it on. Overall, our method is more effective in CT image sequence restoration than the alternative methods.

## Conclusion and Prospect

This paper proposed image sequence restoration for CT scan image using Wiener filtering, based on sparse and low-rank decomposition. One of the key points to this method is uniting the CT image restoration under the framework of low-rank models: we first decompose the CT image sequence into sparse component and low-rank component in the RPCA model if noise level is low, and then restore both the two components using wiener filtering; in the presence of high level noise, it is better to select the GoDec model to decompose the images into three components, namely a sparse component, a low-rank component and a noise component, and then restore the sparse component and low-rank component using wiener filtering. It takes about 1.5s for our algorithm to recover a CT image of size 512
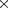
512. Experiments demonstrated that our method generated better results, compared with the other methods. In terms of medical CT image sequence restoration, our method is highly practical, with clear organ boundary and detailed information in the recovered image. The recovered images also have good soft tissue visibility and image quality. One limitation of our method is the reliance on matrix decomposition: in particular, if the low-rank component of every image in the sequence has a large variation, then our method does not work well. In this paper, both the parameters of the blur kernel and the noise-signal ratio of wiener filtering take empirical values, in the future work, we will seek other paths to determine these parameter values automatically and to explore more accurate blur kernels for the CT images.

## References

[1] Hussien MN, IqbalSaripan M (2010) Computed Tomography Soft Tissue Restoration using Wiener Filter. Proceedings of 2010 IEEE Student Conference on Research and Development, 13–14 Dec Putrajaya, Malaysia.

[2] Jiang M, Wang G, Skinner MW, Rubinstein JT, Vannier MW (2003) Blind deblurring of Spiral CT image. IEEE Trans. Med. Imaging 22: 251–262. 10.1109/TMI.2003.815075 12906237

[3] Wang J, Wang G, Jiang M (2005) Blind deblurring of spiral CT images Based on ENR and Wiener filter. IOS Press Journal of X-Ray Science and Technology 13: 49–60.

[4] Liu WB (2007) Blind deblurring algorithm of CT image based on edge to noise ratio and constrained least squares [D]. Peking University, BeiJing.

[5] Al-Ameen Z, Sulong G, Johar MGM (2012) Fast Deblurring Method for Computed Tomography Medical Images Using a Novel Kernels Set. International Journal of Bio-Science and Bio-Technology 4(3): 9–19.

[6] Xu YQ, Hu XY, Wang L, Peng S (2012) Single Image Blind Deblurring with Image Decomposition. IEEE, ICASSP.

[7] Fazel M, Hindi H, Boyd S (2001) A rank minimization heuristic with application to minimum order system approximation. Proceedings American Control Conference 6: 4734–4739.

[8] Cai JF, Ji H, Liu CQ, Shen ZW (2012) Framelet based blind motion deblurring from a single image. IEEE Transaction on Image Processing. 21(2): 562–572. 10.1109/TIP.2011.2164413 21843995

[9] Cai JF, Osher S, Shen ZW (2009) Split Bregman method and frame based image restoration. Multiscale model, Simul 8(2): 337–369.

[10] Fergus R, Singh B, Hertzmann A, Roweis ST, FreemanWT (2006) Removing camera shake from a single photograph. ACM Transactions on Graphics 25, 787–794.

[11] Wen CY, Lee CH (2002) Point spread functions and their applications to forensic image restoration. Forensic Science Journal 1: 15–26.

[12] Jiang M, Wang G (2003) Development of blind image deconvolution and its applications. J. X-ray Science and Technology 11 (1): 13–19. 22388094

[13] William KP, Faramarz D (1997) Fast computational techniques for Pseudo inverse and Wiener image restoration. 06.

[14] Hu XP, Chen GL, Mao ZY (2007) Study on Wiener filtering for restoration of defocus blur image. Chinese Journal of Scientific Instrument. 28(3).

[15] Yuan XM, Yang JF (2009) Sparse and low-rank matrix decomposition via alternating direction method. Available: http://math.nju.edu.cn/~jfyang/files/LRSD-09.pdf. Accessed 2013 Aug 12.

[16] Xing B, Wang J (2012) Denoising of Medical CT Image Based on Sparse Decomposition. Journal of Biomedical Eng 29(3): 456–459. 22826939

[17] Chen S, Donoho D, Saunders M (2012) tomic decomposition by basis pursuit. SIAM Journal on Scientific Computing 29(3): 456–459.

[18] Fazel M (2002) rank minimization with applications. PhD thesis, Stanford University.

[19] Lin ZC, Chen MM, Ma Y (2011) The Augmented Lagrange Multiplier Method for Exact Recovery of Corrupted Low-Rank Matrices. Available: http://arxiv.org/abs/1009.5055. Accessed 2013 Aug 12.

[20] Bertsekas D (1982) Constrained Optimization and Lagrange Multiplier Method. Waltham, MA: Academic Press.

[21] Bertsekas D (1999) Non-linear Programming. Nashua, NH: Athena Scientific.

[22] Wright J, Peng Y, Ma Y, Ganesh A, Rao S (2009) Robust principal component analysis: Exact recovery of corrupted low-rank matrices via convex optimization. Available: http://perception.csl.illinois.edu/matrix-rank/Files/nips2009.pdf. Accessed 2013 Aug 12.

[23] Lin ZC, Liu RS, Su ZX (2011) Linearized Alternating Direction Method with Adaptive Penalty for Low-Rank Representation. NIPS, Optimization and Control.

[24] Cai J, Candès E, Shen Z (2008) A singular value thresholding algorithm for matrix completion. Available: http://arxiv.org/abs/0810.3286. Accessed 2013 Aug 12.

[25] Lin Z, Chen M, Wu L, Ma Y (2009) The augmented Lagrange multiplier method for exact recovery of corrupted low-rank matrices. UIUC Technical Report UILU-ENG-09-2215; arxiv:1009.5055.

[26] Liu G, Lin Z, Yu Y (2010) Robust subspace segmentation by low-rank representation. In Proceedings of the 27th International Conference on Machine Learning, Haifa, Israel.

[27] Tao M, Yuan XM (2011) Recovering low-rank and sparse components of matrices from incomplete and noisy observations. SIAM Journal on Optimization 21(1): 57–81.

[28] Yang J, Yuan X (2011) Linearized augmented Lagrangian and alternating direction methods for nuclear norm minimization. Mathematics of Computation 82: 301–329.

[29] Zhou TY, Tao DC (2011) GoDec: Randomized Low-rank & Sparse Matrix Decomposition in Noisy Case. Proceedings of the 28^th^ International Conference on Machine Learning, Bellevue, WA, USA.

[30] Bredies K, Lorenz, D A (2008) Iterated hard shrinkage for minimization problems with sparsity constraints. SIAM Journal on Scientific Computing 30(2): 657–683.

[31] Cai J, Candès EJ (2010) ShenZ (2010) A singular value thresholding algorithm for matrix completion. SIAM Journal on Optimization 20(4): 1956–1982.

[32] Cheng L, Gong M, SchuurmansD, Caelli T (2011) Real-time discriminative background subtraction. IEEE Trans on Image Processing 20(5): 1401–1414. 10.1109/TIP.2010.2087764 20959270

[33] Donoho DL (2006) Compressed sensing. IEEE Trans. on Information Theory 52(4): 1289–1306.

[34] Zhou T, Tao D (2011) Bilateral random projection based low-rank approximation. Technical report.

[35] Tohnak S, Mehnert AJH, Mahoney M, Crozier S (2011) Dental CT metal reduction based on sequential substitution. Dentomaxillofacial Radiology 40: 184–190. 21346086 10.1259/dmfr/25260548 PMC3610941

[36] Dong J, Kondo A, Abe K, Hayakawa Y (2011) Successive iterative restoration applied to streak artifact reduction in X-ray CT image of dento-alveolar region. Int J CARS 6: 635–640. 10.1007/s11548-010-0544-2 21207177

[37] Aftab K, Yin H (2012) Quality Measure for Blind Image Deblurring. Imaging Systems and Techniques (IST), 2012 IEEE International Conference on Date of Conference: 16–17 July 2012.

[38] Hufnagel RE, Stanley NR (1964) Modulation Transfer Function Associated with Image Transmission through Turbulent Media. Journal of the Optical Society of America 54(1): 52–61.

[39] Wang Z, Bovik AC, Ligang L (2002) Why is image quality assessment so difficult? Acoustics, Speech, and Signal Processing,2002 Proceedings 14: IV–3313-IV-3316.

[40] Zhou JL, Hang Lv (2001) Image enhancement based on a new g0065netical algorithm. Chinese journal of computers 24(9): 959–964.

[41] Rosenfeld A, Avinash C K (1982) Digital Picture Processing[M]. NewYork:Academic Press. 154–167.

